# Divides of identity in medicine and surgery: A review of duty-hour policy preference

**DOI:** 10.1016/j.amsu.2020.07.006

**Published:** 2020-07-10

**Authors:** Connor T.A. Brenna, Sunit Das

**Affiliations:** a Faculty of Medicine, University of Toronto, Toronto, ON, Canada; b Division of Neurosurgery, University of Toronto, Toronto, ON, Canada; c Centre for Ethics, University of Toronto, Toronto, ON, Canada

**Keywords:** Residency, Duty hours, iCOMPARE trial, FIRST Trial

## Abstract

Surgery and Medicine are broadly considered as the two fundamental paths that a physician's career can follow. But their convergence under the singular umbrella of doctoring is relatively recent in the context of medical history. Their co-existence within the structure of medical education and the healthcare system suggest that they bear great similarity to each other, when in reality several differences are intuitively recognizable between them. Here, we discuss recent evidence suggesting a discrepancy between these two streams in the work-hour policy preference of trainees. We argue that these differences betray a more radical divide between them, and one which illuminates an essential difference in the self-identification of surgical and non-surgical medical trainees. Additionally, these findings support a novel claim about the importance of uninterrupted relationships on the formation of professional identity among healthcare professionals. We suggest that the principal separation of surgical and non-surgical practice is significant enough to reconsider their dogmatic unification as well as warrant the adoption of unique rules and policies to govern each stream.

The medical system houses many professional identities: while all are united in the identity of “doctor,” physicians necessarily bear membership to their individual fields and establish their own perception of “self” in relation to it [1]. For clinicians at any stage of practice, a strong professional identity can positively or negatively influence interactions with others and the development of an individual ethic [2]. The processes by which these identities are believed to form are broadly drawn and continuous, beginning with (or perhaps even before) undergraduate medical education [3] and our experiences as resident-physicians [4], but then continuing throughout professional life [5].

Medicine and Surgery exist in parallel as the two archetypical streams of medical education and practice. Despite known and intuitive differences between these two families, Medicine and Surgery are widely considered to be overlapping variations of the same essential skills and services. They share in common many of the fundamental tenets and goals of medical practice: to create robust therapeutic relationships [6], to achieve diagnosis by assessing diagnostic probability and enhancing it with information specific to an individual patient [7], and—each in their own way—to treat pathology and restore or maintain good health. The structure of undergraduate medical education manifests this sentiment: while the aim of most professional training programs is to prepare a diverse incoming student cohort to graduate with a discrete skill set that is practical and applicable to one specific profession, medical schools uniquely admit students on the basis of a single set of criteria (a combination of elements such as academic history, admissions tests, personal statements, and interviews, each graded with the same key and bearing uniform weight for all applicants [8]), offer them the same foundational education, and then prepare them to graduate into a breadth of disparate fields. The modern medical school graduate is positioned to choose from dozens of unique post-graduate training programs, most followed by the opportunity for further subspecialization [9].

Despite their commonalities, the history of Medicine and Surgery does not reflect any suggestion of phylogenetic similarity and, in fact, they do not even share common ancestry. Traditionally, these professions were radically estranged [10]. Surgery—likely because of the physicality of its practice—was considered a trade, and learned by apprenticeship rather than in universities [10]. Only in the 18th century did its practice depart from the traditional imagery of “butchering” barber surgeons, and it converged in both training pathways and workplace spheres with the medical doctors of the day [10]. A vestige of this bifurcation persists in several regulatory bodies bearing the composite title of “College of Physicians and Surgeons”. However, despite the merger of these two fields (more than two hundred years ago), Medicine and Surgery still manifest significant and enduring differences which continue to influence notions of professionalism for practitioners of each.

## 1 Divides between Medicine and Surgery

Intuitively, we can recognize several contrasting features between Medicine and Surgery, spanning from differences at the trainee level (such as training program size and call coverage) to issues that continue on to professional life (such as the nature of patient interactions and coordination of ambulatory and hospital-based patient care). Another interesting observation is that, in many medical fields, the fundamental roles of diagnosis, patient care, and (procedural) intervention are often segregated (e.g. diagnostic versus interventional cardiology) [[11], [12], [13]]. In contrast, these roles are amalgamated in many surgical fields, as embodied in the surgeon who operates in the morning and runs a clinic in the afternoon.

Work hours, similarly, are known to vary significantly between interventional and non-interventional specialties: while the former are not limited to the field of Surgery, Surgery as a field is by definition interventional [14]. This dichotomy is particularly severe during residency, where procedural exposure in medical training is often limited to those pursuing fellowship training [[15], [16], [17]]. A systematic review by Peel et al. identified three key factors in medical graduates’ decisions to pursue a career in a surgical discipline, rather than a non-surgical one, supporting the claim that the streams are in some way unique: gender (an umbrella term including gender discrimination, a perceived deterrence to a career in surgery for women, gender bias in career advancement, lack of female role models, and the impact of parenting and lifestyle considerations), the availability of surgical exposure and training in medical education, and—most heavily studied—the lifestyle and perception of “work-life balance” of a career in surgery [18]. These differences speak to the continued need for adaptation in surgical training programs, particularly with respect to addressing the ongoing issue of gender as a barrier to pursuing a surgical career [19,20]. But they also contribute to the foundation of an argument that surgical and non-surgical specialties are foundationally distinct.

Recent data on perceptions of duty-hour policies in post-graduate medical and surgical trainees support this hypothesis. The iCOMPARE Trial randomly assigned participating internal medicine programs in the United States to one of two groups: one which followed standard duty-hour policies (specifying limits on shift length and mandatory time off between shifts), and one which followed flexible duty-hour policies (no hard rules restricting time spent at work) [21]. As measured by examination scores, the divergence in duty hour restrictions had no significant effect on learning. However, medicine interns in flexible duty-hour programs were less satisfied with their educational experience than their peers in programs randomized to standard duty-hour policies (interestingly, in opposition to the opinions expressed by their program directors) [21,22]. Conversely, interns randomized to standard duty-hour policies reported a greater dissatisfaction in the continuity of care they were observing, compared to their peers in flexible duty-hour programs. Overall, medicine interns largely favoured inelastic standard duty-hour policies that would strictly limit the length of shifts [21].

The FIRST Trial performed an analogous, national cluster-randomized study of duty-hour flexibility in general surgery residency programs in the US [23]. Interns were again randomly assigned to standard or flexible duty-hour policies. Flexibility in the work hour restrictions for surgical programs often manifested as decision-making by interns to stay to the completion of a surgical case, and FIRST demonstrated that these trainees perceived greater availability for both elective and urgent cases [23]. As in iCOMPARE, FIRST found no difference between the two groups in terms of clinical outcomes, specifically, in patient mortality or primary/secondary patient complications. In contrast to findings from iCOMPARE, however, surgical interns from programs in FIRST following flexible duty-hour policies were found to be less likely to report dissatisfaction with patient safety, continuity of care, professionalism, or resident education, compared to their peers from programs randomized to standard duty-hour policies. These gains came at a cost: interns in flexible duty-hour programs were more likely to report perceiving negative effects on their personal activities outside of work than their peers from standard duty-hour programs [23]. Nonetheless, surgical residents reported a preference for flexible policies with increasing frequency as they progressed temporally through their training programs, as well as a sentiment that rigid duty-hour policies were negatively affecting critical outcomes like patient safety, morale, and continuity of care [24].

In both the FIRST and iCOMPARE Trials, surgical and medical residents working in systems with flexible duty-hour policies reported less satisfaction with their own well-being, but greater satisfaction with the continuity of patient care they were providing, compared to their interns from programs randomized to standard duty-hour policies [[21], [22], [23]]. In contrast to their medical peers, however, surgical residents broadly supported these policies and the ideology that they should have the flexibility to stay longer at work (evidencing a preference for the option to participate in an interesting case in the operating room or to finish handling an active patient issue on the ward), while medical residents supported policies that would put hard caps on their hours in the hospital [22]. These two groups of residents, having matriculated from the same undergraduate medical system after entering medical school through analogous admissions processes, seem to *want* something essentially different.

## 2 A fundamental difference in values

The discrepancy in duty-hour policy preference suggests a principal distinction between Surgery and Medicine training programs, either intrinsic to the trainees in these programs or to the nature of the programs within which they exist. There are many possible explanations for the discordance. One postulate for consideration is that medical graduates who self-select to surgical residencies are inclined toward personality traits that diverge from those that are predominant in medical graduates who are attracted to medical programs, and that the surgical training system (which contains abundant rewards only internal to itself, such that external motivators become undervalued) exacerbates these differences [24]. Alternatively (or additionally), a second possible explanation is that in Surgery—more than in Medicine—there is no reliable replacement for case volume as a means toward achieving proficiency, a finding that trainees increasingly appreciate as they proceed further along their training. In other words, the ethos of Surgery may fundamentally differ from that of Medicine because of the high-stakes demand that its graduates be capable of independently performing what they have learned. Finally, a third possible explanation is that the personal drivers of those graduates who pursue a career in Surgery are principally unique from those of graduates pursuing a career in Medicine. Any of these cases would constitute evidence in support of the premise that there is a fundamental and meaningful difference in the value systems maintained by trainees in each stream.

## 3 The role of patient relationships in identity formation

The divergence of values delineated by the iCOMPARE and FIRST trials suggest that the professional identities constructed by medical and surgical trainees are distinct. We propose that this divergence in our understandings of professionalism—and how we think of ourselves as physicians—both influences and is mediated by our choice of profession. To support this claim, we put forth an additional and novel argument: that uninterrupted relationships with patients are particularly critical for the formation of both a personal and professional identity in surgery, and that surgical trainees prefer flexible work-hour policies—even while acknowledging their personal cost—because these measures allow them to cultivate these relationships.

The process of declaring oneself as a patient's caretaker—and the act of assuming the responsibilities that come with this declaration—are vital to the development of the patient-doctor relationship. This process is just as critical an element to the genesis of a professional identity. Professional identity formation during medical training requires trainees to have opportunities to assume responsibility for, and ownership over, patient care—in other words, to have opportunities to exercise autonomy within the scope of their chosen identity [25]. The value of continuity of care to quality of care has been well documented [26]; we propose that it is just as critical to the social and professional development of those who are providing that care. Whether or not we require uninterrupted relationships to develop our physical skills as medical professionals, we do need them to form our identities as physicians, and to sustain us through the journey of medical education and practice.

The demands created by the episodic nature of surgical care necessarily differentiate the nature of relationships in Surgery from those in Medicine. Surgical cases do not adhere to the inflexible boundaries of work-hour policies. For example, a trainee working overnight may admit a patient in critical circumstances with plans made for surgery in the coming day, after her shift has ended. Proponents of strict work-hour restrictions will appropriately note that the case missed by an intern today will be compensated by the case handed off to her tomorrow. However, neither she nor her colleague will have access to the benefits of the uninterrupted relationship each could otherwise form with a patient by providing their care from start to finish. This process of assessment, developing a plan, building trust, and execution—in other words, of taking complete ownership over the care of a surgical patient—is critical to the development of professional identity as a surgeon (just as it may be for non-surgical trainees). While it could be argued that this tension will resolve itself over the timescale of a training program by means of trainees piecing together an idea of ownership from each of its constitutive parts, experienced with different patients on different days, the result is a plausible facsimile rather than the actual experience of patient care. It is appropriate to recall the weight of this loss within the context of the personal burden of work demands on trainees [27]. But the weight of this loss should be considered.

The relationships that trainees form with patients for whom they provide care are critical to the process of becoming a doctor—in surgical and non-surgical fields. But the benefits of contiguous long hours differ for surgical trainees, and boundaries to clinical opportunities may unintentionally discredit those relationships for them. The discrepancy in work-hour policy preference among trainees within the two streams of Surgery and Medicine may represent an implicit understanding among surgical trainees that inflexible work-hour boundaries negate opportunities otherwise available to them to develop a relationship with a patient that includes shepherding her through her whole episode of need: taking ownership over her in the emergency department, forging a therapeutic connection with her, participating in her operation, continuing her therapeutic connection following surgery, and discharging her from the hospital.

## 4 Embracing division

Based of these findings, we posit that the historical divide between Surgery and Medicine may not have been entirely unwarranted. In many ways the two families of practice, although they share a common pathway in undergraduate medical education, have an underlying divergence that has been largely underplayed in their merger. Since these streams were united under a common title, we as one profession have in the policies and institutions of medicine as a greater whole coupled them together with a final goal of constructing training programs that enable trainees to reach then end of competency. However, trainees within these two streams appear to be in need of—or privileging—different means, suggesting that there is a greater difference in values between each group than there is within each group. The data suggest that the need for uninterrupted patient relationships in the development of professional identity also illuminates an asymmetry between the fields of Surgery and Medicine, which is not supported by identical policies of governance. In the eyes of those trainees for whom work-hour policies are supposedly created to protect, these streams of postgraduate medical education are not equivalent.

This viewpoint becomes obscured in the estuaries where both streams interface within single individuals: for example, critical care clinicians who have undertaken surgical training, or interventional practitioners within a medical subspecialty. We do not claim that the dichotomy between these fields is absolute. At the less granular level of Surgery and Medicine, however, we suggest that these families of practices are more inherently divided than current models of medical education and industry would suggest. Implied in this claim is an argument that a divergence in the rules and policies governing surgical and non-surgical streams of training and practice—borne out of a respect for their essential differences—will allow for each to perform more efficiently, alongside as well as independent of the other.

## Provenance and peer review

Not commissioned, externally peer reviewed.

## Funding

The authors have no funding to acknowledge in support of the submitted manuscript.

## Ethical approval

N/A.

## Consent

N/A.

## Author contribution

Connor Brenna performed a review of the pertinent literature and wrote the paper. Sunit Das was responsible for conceptualization of the study question and edited the paper.

## Registration of research studies

1. Name of the registry: N/A.

2. Unique Identifying number or registration ID: N/A.

3. Hyperlink to your specific registration (must be publicly accessible and will be checked): N/A.

## Guarantor

The Guarantor is the one or more people who accept full responsibility for the work and/or the conduct of the study, had access to the data, and controlled the decision to publish.

Sunit Das.

## Declaration of competing interest

The authors disclose no conflicts of interest relevant to the submitted manuscript.
